# Supplementary material for: Current Clinical Paradigm and Therapeutic Advancements in Thymic Malignancies: A Narrative Review
Source: Cancers (Basel). 2025 Nov 11;17(22):3622. doi: 10.3390/cancers17223622 (PMC12650598; doi:10.3390/cancers17223622)
Supplement: Supplementary file 1 [file cancers-17-03622-s001.zip › cancers-3898013-supplementary.pdf]

Supplementary Table S1. WHO Classification of Thymoma	
**Thymoma**	
**Type A**	Spindle- or oval-shaped epithelial cells with bland, relatively uniform cytology and minimal to absent immature lymphocytes.
**Type AB**	Biphasic tumor showing areas resembling Type A thymoma admixed with nodules or regions rich in immature lymphocytes.
**Type B1**	Recapitulates normal thymic cortex, with abundant immature T-lymphocytes and scattered, inconspicuous epithelial cells, often with small medullary-like foci.
**Type B2**	Dense infiltrate of immature lymphocytes with more conspicuous epithelial cells forming clusters or delicate networks and mild cytologic atypia.
**Type B3**	Predominantly epithelial neoplasm composed of polygonal cells with mild-to-moderate atypia and only sparse lymphocytes.
